# Laser-Synthesized 2D-MoS_2_ Nanostructured Photoconductors

**DOI:** 10.3390/mi14051036

**Published:** 2023-05-12

**Authors:** Igor A. Salimon, Ekaterina V. Zharkova, Aleksandr V. Averchenko, Jatin Kumar, Pavel Somov, Omar A. Abbas, Pavlos G. Lagoudakis, Sakellaris Mailis

**Affiliations:** 1 Center for Photonic Science and Engineering (CPhSE), Skolkovo Institute of Science and Technology, 3 Nobel Street, 143026 Moscow, Russia; igor.salimon@skoltech.ru (I.A.S.); ekaterina.zharkova@skoltech.ru (E.V.Z.); aleksandr.averchenko@skoltech.ru (A.V.A.); jatin.kumar@skoltech.ru (J.K.); o.abbas@skoltech.ru (O.A.A.); p.lagoudakis@skoltech.ru (P.G.L.); 2 Center for Energy Science and Technology (CEST), Skolkovo Institute of Science and Technology, 3 Nobel Street, 143026 Moscow, Russia; pavel.somov@skoltech.ru

**Keywords:** 2D materials, transition metal dichalcogenides, direct laser writing, laser induced periodic surface structures (LIPSS), laser synthesis of materials, persistent photoconductivity

## Abstract

The direct laser synthesis of periodically nanostructured 2D transition metal dichalcogenide (2D-TMD) films, from single source precursors, is presented here. Laser synthesis of MoS_2_ and WS_2_ tracks is achieved by localized thermal dissociation of Mo and W thiosalts, caused by the strong absorption of continuous wave (c.w.) visible laser radiation by the precursor film. Moreover, within a range of irradiation conditions we have observed occurrence of 1D and 2D spontaneous periodic modulation in the thickness of the laser-synthesized TMD films, which in some cases is so extreme that it results in the formation of isolated nanoribbons with a width of ~200 nm and a length of several micrometers. The formation of these nanostructures is attributed to the effect that is known as laser-induced periodic surface structures (LIPSS), which is caused by self-organized modulation of the incident laser intensity distribution due to optical feedback from surface roughness. We have fabricated two terminal photoconductive detectors based on nanostructured and continuous films and we show that the nanostructured TMD films exhibit enhanced photo-response, with photocurrent yield increased by three orders of magnitude as compared to their continuous counterparts.

## 1. Introduction

Two-dimensional materials are ultrathin crystalline layers that bond to each other by van der Waals forces and therefore single layers have been isolated, starting from the Nobel prize-yielding work on graphene [1], which is a monolayer of graphite. Among 2D materials there exists a subclass of semiconducting layered crystals called transition metal dichalcogenides (TMDs) [2] that have attracted much interest lately due to a plethora of interesting physical properties, spanning from optical nonlinearity [3] and magnetism [4] to superconductivity [5], to name a few, which stems from their compositional tuneability by changing the combinations of transition metals and chalcogens to achieve the desired electrical and optical properties. Consequently, much research has been conducted in recent years on various aspects of the synthesis and utility of these compounds to investigate the length and breadth of their capability to be exploited in cutting edge technological applications such as optoelectronics, sensing, and quantum information [6]

Recently, we have demonstrated that it is possible to employ a laser-based method to synthesize in situ MoS_2_, WS_2_ [7], which are among the most extensively studied TMDs as well as their alloys (Mo_x_W_1−x_S_2_) [8]. This laser-based synthesis method relies on localized thermal decomposition of single-source precursors (a Mo and/or W thiosalts) [9,10] induced by a focused laser beam on a precursor film. Interestingly, laser synthesis of TMDs occurs in ambient conditions and with a minimum thermal load on the substrate, making it suitable for additive manufacturing. Furthermore, it has been shown that the local nature of the laser synthesis process leads to readily micro-structured films, which provides another level of convenience to the ease of synthesis in device fabrication, as it simplifies the fabrication process for electronic or photonic devices. Indeed, the films are synthesized along tracks that have widths that are comparable to the width of the focused laser beam that was used for the localized thermal dissociation. Continuous films with much larger areas can be formed by stacking together adjacent laser-synthesized TMD tracks. 

It has been reported that nano-structuring of 2D-TMD has the potential to improve the performance of these materials in applications such as switching and sensing [11]. For instance, Daus et al. demonstrated a flexible monolayer MoS_2_ FET with a channel length of 60 nm, which possesses ultra-high on-current (~500 µA) at a very low gate voltage (1V) [12]. Furthermore, a superior photosensitivity (2.67 × 10^6^ A/W at λ = 520 nm) has been demonstrated by applying nanobridge technology to fabricate multi-nanoheterojunctions MoS_2_ phototransistors [13]. 

Here, we present the utilization of another structuring tool, which is associated with the use of a laser source in the TMD film manufacturing process and produces sub-micron patterning along or across the laser-synthesized TMD film tracks. This ultra fine patterning tool is based on an optical effect that is known as laser-induced periodic surface structures (LIPSS). This is a universal effect that is observed under certain conditions in laser writing experiments [14] and originates from the interaction between the incoming laser radiation with secondary waves that stem from scattering by surface roughness. The result is a self-organized regular spatial modulation (1D or 2D) of the local laser intensity, which in turn modulates the interaction with the surface, resulting typically in a regular modulation of the surface topography [15]. The modulation of the topography can be caused by material removal (laser ablation), where variations of the local intensity register in the amount of material that is removed, and thus the resulting topography reflects the optical intensity modulation [16]. Another way of modifying the surface topography is by laser-induced photochemical/thermochemical processes such as local oxidation [17,18]. LIPSS consists typically of periodic features, with a period that is comparable to the wavelength of the irradiating laser, which are oriented in accordance with the polarization of the incident beam [14].

It is shown here that 1D and 2D LIPSS patterns are observed during the laser synthesis of TMDs, manifesting themselves as a modulation of the thickness of the TMD film. The orientation of these periodic features with respect to the track depends upon the polarization of the laser beam, and in some cases the thickness modulation is so extreme that it produces isolated ribbons with a width of ~200 nm. Furthermore, noting that such extreme nano-structuring of 2D materials can introduce new effects/capabilities, we have employed MoS_2_ nanoribbon arrays in the production of photodetectors with a performance that surpasses their unmodulated counterparts by several orders of magnitude. LIPSS nano-patterning has been observed in the synthesis of both MoS_2_ and WS_2_, which is not surprising given the universal nature of the effect. Here we will be presenting primarily results that correspond to MoS_2_. Evidence for 1D LIPSS formation in the synthesis of WS_2_ (SEM imaging and Raman spectroscopy) is given in Figure S1 of the Supplementary Information.

## 2. Materials and Methods

The laser synthesis of MoS_2_, WS_2_, and alloy films (of various compositions) is described in detail in [7,8]. We will, however, outline the process here for clarity. It consists of three step in the following manner. Step one: a solution of the precursor in a system of organic solvents is spin-coated onto SiO_2_/Si substrates and then allowed to dry at a temperature of ~90 °C for 5 min. Step two: the dried precursor film is irradiated locally using a focused laser beam in the visible wavelength range (here we used a c.w. laser emitting at λ = 532 nm), which results in the synthesis of TMD tracks. Step three: the laser-irradiated sample is “developed” by immersion in organic solvents, which remove the unexposed precursor, revealing only the synthesized TMD tracks. 

### 2.1. Substrate Preparation

Wafers of n-type silicon with a 300 nm oxide layer were diced into 1.5 cm^2^ squares that were used as substrates for the TMDs. The substrates underwent cleaning by ultrasonication in acetone, isopropanol, and then deionized water in this order. After the deionized water cleaning step, followed by drying using nitrogen flow, they underwent plasma etching in a mixed argon and nitrogen atmosphere for 15 min to remove any traces of organic contaminants and to produce a hydrophilic surface that improves the coverage during the spin coating step.

### 2.2. Precursor Film Preparation

The concentrations of the ammonium tetrathiomolybdate ((NH_4_)_2_MoS_4_) precursor solutions that were used here were: 24, 32, 40, and 48 mM. The system of organic solvents that was used to dissolve (NH_4_)_2_MoS_4_ consists of dimethylformamide (DMF), butylamine, and ethanolamine (at volumes of 2, 2, and 1 mL, respectively). The precursor solution was sonicated for 40 min. The resulting precursor solution was spin-coated on the substrates. The top speed of spin coating was 3500 rpm with a ramp speed of 500 rpm/s. The total duration of the spin coating was 1 min. After spin coating, the film was dried on a hot plate for 5 min at a 90 °C to remove the organic compounds. 

### 2.3. Laser Irradition

A continuous wave (c.w.) 532 nm laser system, Coherent-Verdi G20, was used to irradiate the precursor films. The laser beam was focused to a spot with a diameter of ~11 μm, using a microscope objective (×4). A high-precision x–y translation stage, Aerotech ANT95XY, was used to move the substrate below the focused laser spot, thus exposing preferentially different areas of the film. In the experiments that are presented here, the laser power was within the range of 400–950 mW while the scanning speed (the linear velocity of the translation stage) was within the range of 1–20 mm/s. The laser irradiation process took place in *ambient conditions*. The parameters of laser synthesis for the samples used in the figures are given in the Supplementary Information (Table S1). The polarization of the incident laser beam was linear and perpendicular to the scanning direction, unless otherwise stated. After laser irradiation, the samples were “developed” in n-methylpyrrolidone (NMP) or in DMF to remove unexposed areas of precursor film.

### 2.4. Characterization

Samples were analyzed by means of Raman spectroscopy, atomic force microscopy (AFM), and scanning electron microscopy (SEM). The LabRAM HR Evolution (HORIBA) system was used to conduct Raman spectroscopy. AFM was performed by the NTMDT-SMENA to investigate the topography of the laser scan tracks. SEM images were obtained using Tescan Solaris dual-beam scanning electron microscope (Tescan, Brno, Czech Republic) and Quattro S scanning electron microscope (ThermoFisher Scientific, Waltham, MA, U.S.). Electrical measurements were carried out using a B1500A Agilent semiconductor analyzer.

### 2.5. Photodetector Device Fabrication

Photodetectors were fabricated from single tracks of laser-synthesized MoS_2_ with a channel length (distance between contacts) of ~5 μm and a width of ~200 μm. Gold contacts were deposited on either side of the track (as shown in Figure S2 of the Supplementary Information) where the central region of the MoS_2_ track is located in the gap between contacts. The contacts were pre-defined using UV laser photolithography and double layer photoresist (an AZ1505 positive photoresist was deposited on an LOR5B sublayer) to optimize the lift off process, followed by 40 nm gold film magnetron sputtering deposition and lift-off. 

## 3. Results and Discussion

Direct laser synthesis of MoS_2_ produces tracks of thin films with a topography that is associated with the irradiating laser beam intensity profile at focus. This is due to the variable temperature profile, which is induced by the intensity variation within the laser spot. In the experiments described here, the laser beam (λ = 532 nm) had a Gaussian intensity profile, which produced films with a variable thickness in the direction perpendicular to that of the track due to the non-uniform intensity profile of the beam. Thus, the thinnest part of the film was at the center of the track, while the thickness increased gradually towards the periphery. Raman spectroscopy revealed that the MoS_2_ material was only formed in the central section of the track, while the thicker parts probably consisted of amorphous MoS_3_, which can also be synthesized from the same precursor at lower temperatures [19]. To illustrate the thickness variation across the track, an AFM image of a single MoS_2_ track is shown in Figure S3 of the Supplementary Information.

**Figure 1:**
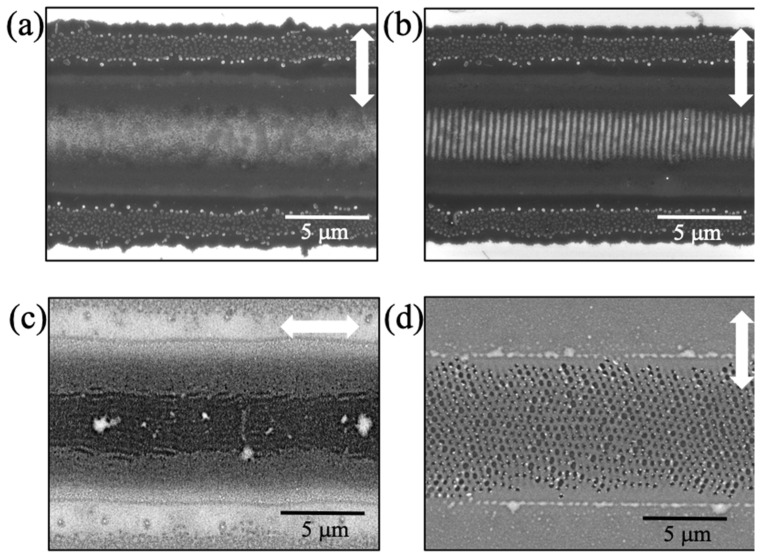
SEM images showing (**a**) continuous MoS_2_ film; (**b**) film with periodically modulated thickness (1D LIPSS); (**c**) 1D LIPSS formed with beam polarization oriented along the direction of scanning; and (**d**) 2D LIPSS. The white arrows indicate the beam polarization. The track runs along the horizontal direction.

Operating within the range of the experimental parameters, which have been outlined in the Methods section, we have observed the formation of 1D and 2D LIPSS features that are mainly associated with the central region of the track (that corresponds to the peak of the intensity) and propagate along the length of the track. Figure 1 shows scanning electron microscopy images of a continuous MoS_2_ track (Figure 1a) and one with periodically modulated thickness (Figure 1b). Both tracks were produced on the same sample using the same precursor film by merely varying the laser scanning speed. The polarization of the beam, however, was the same in both cases, as indicated by the white arrows. When the beam polarization was rotated by 90 degrees, the LIPSS features rotated to follow the direction of the polarization (Figure 1c). Finally, at certain conditions of irradiation, as will be discussed in more detail later, 2D LIPSS features were observed (Figure 1d).

The occurrence of 1D and 2D (or both) LIPSS features appears to depend on the thickness of the precursor film, which is determined by the concertation of the precursor in the initial solution. At low concentrations (24 and 32 mM), only 1D LIPSSs were observed. At a precursor concentration of 40 mM, it was possible to observe both 1D and 2D patterns. For 48 mM, only 2D LIPSS could be observed. For intermediate precursor concentrations, where both 1D and 2D features are possible, it was observed that at lower scanning speeds (between 2 and 6 mm/s) only 1D LIPSSs were formed, while for speeds above 16 mm/s only 2D LIPSSs were formed. For the speeds in between, fluctuations in the film could cause random switching between the 1D and 2D patterns.

### 3.1. Linear LIPSS and Formation of Nanoribbons

As shown in the SEM image of Figure 1b, the regular patterns that were formed at lower precursor concentrations consist of linear gratings with a period of 423 ± 9 nm. AFM scans revealed that the periodic features correspond to a modulation of the film’s thickness. This period corresponds to *low spatial frequency* LIPSS [14]. Furthermore, the orientation of the periodic features appears to be aligned with the orientation of the polarization of the irradiating laser beam, which is perpendicular and parallel with respect to the direction of the track in Figure 1b and Figure 1c, respectively.

In the case where the periodic features form perpendicular to the track (as in Figure 1b), the pattern is more regular as compared to the case of Figure 1c where the periodic features form parallel to the direction of the track. Nevertheless, in both cases the period is essentially the same. This qualitative difference is attributed to more efficient optical feedback in the former case [18]. Raman spectroscopy provided verification that both tracks with periodically modulated thickness indeed consist of MoS_2_. Relevant Raman spectra are shown in Figure S4 of the Supplementary Information.

In previous reports [7,8], we remarked that a *laser thinning* process regulates the final thickness of the TMD film, which is associated with the peak intensity of the laser beam. Therefore, we anticipate that it is the variations of the intensity due to optical feedback that lead to the observed thickness variation. 

**Figure 2:**
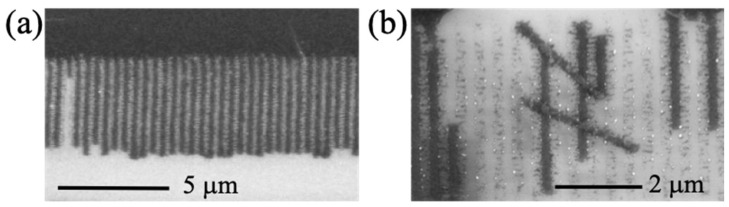
SEM images showing (**a**) the formation of isolated nanoribbons, (**b**) over-developed tracks showcasing detachment and bending of nanoribbons.

Interestingly, for films that were produced at the higher range of intensities and which have been “developed” in organic solvents, the thickness variation was exaggerated to an extent that the grating became a periodic arrangement of isolated ribbons with approximately 200 nm width, as shown in the SEM images of Figure 2. These ribbons were separated by gaps of exposed substrate, which appeared to contain irregular structures of even smaller dimensions (Figure 2a,b) that may be connected to the ribbons. Those smaller structures were about ~50 nm wide and they appeared to be randomly distributed, and therefore they do not qualify as high spatial frequency LIPSS [20,21]. In some cases of excessive “development”, the ribbons may detach from the tracks, as shown in (Figure 2b).

### 3.2. 2D LIPSS

**Figure 3:**
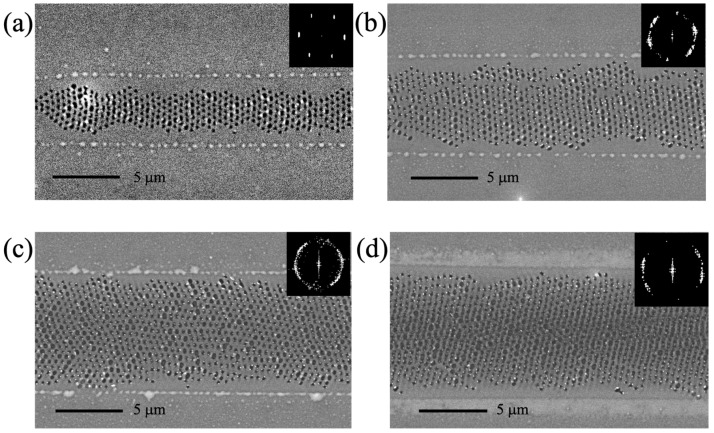
SEM images of 2D LIPSS structures, which have been produced using the different irradiating laser intensities (**a**) 0.51 MW/cm^2^, (**b**) 0.64 MW/cm^2^, (**c**) 0.76 MW/cm^2^, and (**d**) 0.89 MW/cm^2^. The insets correspond to a Fourier transform of the 2D LIPSS pattern.

When denser precursor films are used, 2D LIPSS features start to emerge. These patterns are, as a general rule, produced using faster speeds as compared to their 1D counterparts. Figure 3 presents an overview of 2D LIPSS patterns that have been produced using the same scanning speed, but different laser peak intensities. The width of the 2D LIPSS-covered area scales with the peak intensity value. In addition to that, the regularity of the 2D pattern seems to deteriorate as the intensity of the laser increases, although the separation between features seems to remain the same. This can be seen clearly in the corresponding Fourier transforms of the 2D patters that are shown as insets in each SEM image. The distortion of the 2D pattern that occurs at higher intensities indicates perhaps greater sensitivity to random surface defects. 

Two-dimensional LIPSS in general appear at higher speeds and in denser precursor films, as compared with the 1D LIPSS; however, Raman spectroscopy indicated that in all cases there was successful transformation of the precursor film to MoS_2_. 

**Figure 4:**
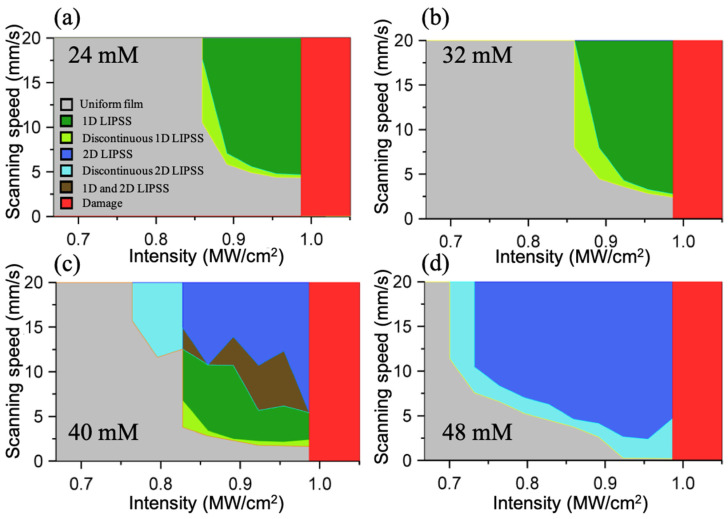
Parameter maps (laser intensity, scanning speed) indicating the regions where various LIPSS formations were observed for (**a**) 24 mM, (**b**) 32 mM, (**c**) 40 mM, and (**d**) 48 mM precursor film concentrations. The color key for the various observations is shown as an inset in (**a**).

A parametric study that yielded these results was conducted to identify the irradiation conditions under which 1D and 2D LIPSSs are formed for various precursor film concentrations. This study was conducted in the following manner: linear tracks of MoS_2_ were produced by laser irradiation, on precursor films with different concentrations. For each track, the laser intensity was fixed but the scanning speed increased linearly with time. Consequently, each position along the laser-synthesized MoS_2_ track corresponds to a specific speed. The areas on the track where different types of LIPSS occur were marked and a map of the parameter space (laser intensity and scanning speed) for the formation of specific structures has been compiled. These parameter maps, corresponding to film concentrations of 24, 32, 40, and 48 mM are shown in Figure 4. Various formations are shown, including 1D-LIPSS (green region), 2D-LIPSS (dark blue region), and intermediate ranges of instability. The parametric study suggests that LIPSS are more likely to form at high speed/high intensity combinations. A higher precursor concentration (corresponding to thicker precursor films) shifts the probability for LIPSS formation to lower intensities and interestingly promotes the occurrence of 2D patterns. At the higher end of laser intensities, the central part of the track is completely removed (red regions). However, periodic features can still be observed in the periphery. 

Scanning electron microscopy investigation of the track topography confirmed literature reports that the LIPSS formation is greatly influenced by the surface topography, which provides optical feedback. For example, we have observed a switch from 1D to 2D LIPSS by encountering a defect in the laser beam path, as shown in Figure S5 of the Supplementary Information.

Importantly, 2D-LIPSS formations dominate in thicker films (40 and 48 mM), whereas 1D-LIPSS dominate in thinner films (24 and 32 mM). The precursor film consists of thiosalt particles, which act as scattering centers for the incident laser beam. Consequently, the amount of scatterers increase with the concentration, suggesting that scattering intensity could be a significant factor in the 1D and 2D LIPSS formation selectivity.

Formation of LIPSS was extensively reported on thin films of metals [18] and polymers [22]. Most works that mention the production of 1D and 2D LIPSS on thin films used pulsed irradiation and demonstrated the dependences of the obtained patterns on the number of laser pulses. Two-dimensional patterns were obtained for circular polarization [23] of the incident beam while linear polarization produced 1D patterns [22]. In the present work, c.w. irradiation with a linear polarization was used to form both 2D LIPSS and 1D LIPSS. Here, the pattern selectivity depends only on the scanning speed and the thickness of the spin-coated precursor film, as defined by the concentration of the thiosalt in the initial solution.

High spatial frequency LIPSS-based patterning of TMDs in pulsed laser thinning experiments [24,25] and on a film of metal-organic precursors used for MoS_2_ synthesis has also been reported [26]. In the latter case, which is relevant to this work, the patterning that was achieved on the precursor film was observed to deteriorate upon conversion to MoS_2_, which renders this approach problematic for practical uses of the nanopatterning. In this work, the patterning is simultaneous with the synthesis of MoS_2_, thus permitting direct study of the impact of nanopatterning in devices such as photodetectors, as will be shown below. 

While various methods of creating structural changes in thin films of MoS_2_ have been reported [27] to achieve patterning that increases the edge to surface fraction, the present study suggests a very simple approach towards obtaining high edge length in surface structures. The use of well-established and available precursor compounds and ambient condition synthesis could make this approach suitable for scalable production or rapid prototyping of nanostructured 2D-TMD.

### 3.3. Nanostructured Photodetectors

Nanostructuring is a promising approach to improve the photoelectrical conversion in 2D materials by engineering the defect states (trap density) within the bandgap, thus enhancing the overall photodetection process. For example, Park et al. [27] proposed a block copolymer lithography nanopatterning method for multilayer MoS_2_ that resulted in a significant increase in photoresponsivity in nanopatterned MoS_2_ films compared to continuous films. Here, we utilized LIPSS-structured MoS_2_, which can form linear arrays of nanoribbons, as a photodetector to study the impact of nanostructuring on the performance of laser-synthesized photodetectors as compared to their continuous counterparts. 

For this purpose, several MoS_2_ tracks were synthesized from a 24 mM precursor film. By adjusting the laser irradiation parameters, 1D LIPSS nano-structured MoS_2_ and continuous film tracks were produced on the same substrate. The photodetectors were fabricated by depositing gold contacts on both sides of the MoS_2_ tracks while the central area of the films served as the photoconductive channel. The device was biased by 5 V and the photocurrent was measured as a function of the optical power at two distinct excitation wavelengths (λ_1_ = 650 nm and λ_2_ = 450 nm). 

**Figure 5:**
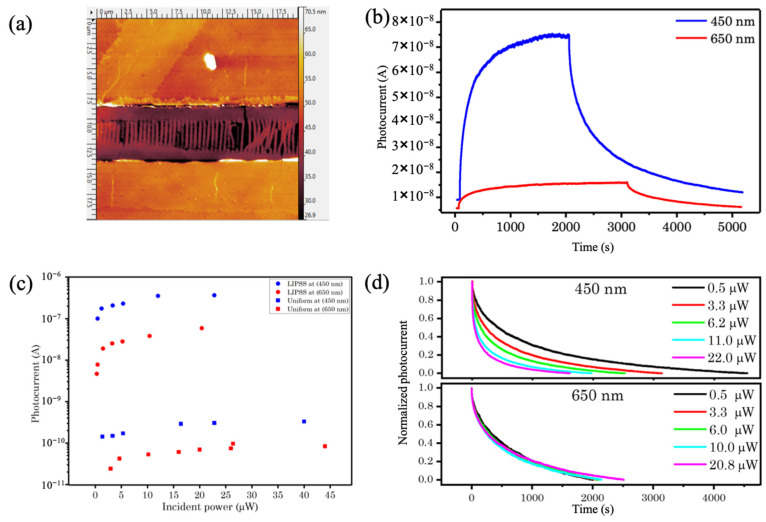
(**a**) AFM image of the nanostructured device. The central section consists of an array of isolated nanoribbons enveloped by a pair of metal (Au) electrodes (top and bottom); (**b**) temporal evolution of photocurrent (growth and decay when excitation laser is switched on and off, respectively) for 650 and 450 nm excitation, at the same power (5.3 µW); (**c**) saturation values of the photocurrent as a function of incident power for 650 and 450 nm excitation, for nanostructured (LIPSS) and continuous devices; (**d**) decay curves of the photocurrent corresponding to 450 nm (top graph) and 650 nm (bottom graph) excitation, for various excitation powers.

Figure 5a shows an AFM image of a section of the nanostructured MoS_2_ photoconductor that consists of a linear array of isolated MoS_2_ nanoribbons that are bridging the gap between the gold contacts. An AFM surface profile along a section of the nanostructured photodetector is shown in Figure S6 of the Supplementary Information.

Figure 5b shows the temporal evolution of the photocurrent for the nanostructured MoS_2_ photodetector obtained using 450 and 650 nm excitations, corresponding to the same optical power level. The rise and fall dynamics of the device are rather slow, suggesting that the device exhibits “persistent photoconductivity” [28,29,30,31]. The saturation value of the photocurrent (at 450 and 650 nm) was measured as a function of illuminating power for both nanostructured and continuous photodetectors and the measurements are summarized in the plot of Figure 5c. Generally, both devices were more responsive to short excitation wavelengths due to higher optical absorption. Most importantly, the amplitude of the photocurrent corresponding to the nanostructured device is three orders of magnitude higher as compared to that measured in the continuous device for both excitation wavelengths.

Considering that both nanostructured and continuous MoS_2_ films were synthesized from the same precursor film using the same laser intensity, this significant enhancement in the photocurrent could be attributed to a substantial increase in the density of shallow traps, as compared to continuous MoS_2_ films, caused by the nano-structuring process [27]. Moreover, surface modification can enhance the absorption of light, e.g., by increasing the overlap between the excitation light and the photoconductor, thus further contributing to the observed photocurrent enhancement.

The photocurrent decay dynamics in nanostructured devices is illustrated in Figure 5d, where the normalized decay curves for nanostructured devices, corresponding to 450 nm (top) and 650 nm excitation are shown. Further analysis of the photocurrent decay curves has been conducted by fitting the double exponential decay function shown below:

$$I_{PC}=A_{1}e^{-\tau_{1}t}+A_{2}e^{-\tau_{2}t}$$

where $I_{PC}$ is the photocurrent and $\tau_{1}$ and $\tau_{2}$ are the fast and slow components, respectively. The fitted values for these decay time constants for 650 and 450 nm are presented Table 1 and Table 2, respectively.

The fitted values for the decay time constants $\tau_{1}$ and $\tau_{2}$ appear to become shorter with increasing laser power at the 450 nm excitation wavelength, whereas they seem to be independent of the excitation power at the 650 nm excitation wavelength. This behavior could be explained by the defect model that is given in [31], which provides a description of the photocurrent dynamics in photoconductors. 

In [31], the photocurrent decay dynamics were associated with the ability to excite preferentially one, or both types of charge carriers, with incident light. In materials that possess a high density of shallow trapping centers in their bandgap it was argued that in a *low intensity excitation regime*, free carriers of one sign are primarily excited. In this case, the photocurrent relaxation dynamics is determined by the relaxation of this type of photocarriers. However, at a *high intensity excitation regime*, photocarriers of both signs are excited, causing a significant increase in the free carrier density. In this case, the photocurrent relaxation dynamics will be faster due to the availability of a multitude of recombination cites. Here, we observed that excitation at 450 nm yields an order of magnitude higher photocurrent as compared to 650 nm. We can therefore attribute the observed dynamics to the difference in the photocarrier excitation efficiency of the two wavelengths, as indicated by the difference in the photocurrent. In these terms, excitation at 650 nm, at the intensity levels that were used in our experiments, corresponds to the *low intensity excitation regime* and therefore there is no intensity dependence of the photocurrent decay times. On the other hand, 450 nm excitation accesses the *high intensity excitation regime* and relaxation time decreases with increasing irradiation power, as described in [31]. 

## 4. Conclusions

We have presented the formation of sub-micron 1D and 2D LIPSS patterns in direct laser-synthesized MoS_2_ film tracks. This regular sub-micron patterning can, in some cases, result in extreme topographical modifications that lead to the formation of arrays of isolated ribbons with widths of ~200 nm and lengths of several micrometers. The resulting nanoribbon arrays exhibits enhanced persistent photoconductivity, and their photocurrent increased by three orders of magnitude compared to their continuous film counterparts. The enhancement in the photocurrent is attributed to the increase of the shallow trapping centers’ density in the film, as a result of the nanostructuring process; thus, LIPSS-based nanopatterning could be a promising route to enhance the photoelectrical properties of TMDs by defect engineering.

## Figures and Tables

**Table 1:** Decay times at 650 nm.

Power (µW)	650 nm Excitation	
	$\tau_{1}$ (s)	$\tau_{2}$ (s)
0.48	99	1055
3.3	107	1104
4.8	112	1134
6.0	134	1228
10.0	108	936
13.2	135	1148
20.8	101	1082

**Table 2:** Decay times at 450 nm.

Power (µW)	450 nm Excitation	
	$\tau_{1}$ (s)	$\tau_{2}$ (s)
0.52	210	1639
1.44	151	1349
3.3	112	1063
5.3	100	931
6.2	95	831
7.0	50	469
11.0	45	457
13.2	32	343
22.0	40	409

## Data Availability

Data will be made available by the authors upon reasonable request.

## Supplementary Materials

The following supporting information can be downloaded at: https://www.mdpi.com/article/10.3390/mi14051036/s1: Table S1: laser scanning parameters; Figure S1: SEM image of LIPSS formation on laser-synthesized WS_2_ track and corresponding Raman spectrum; Figure S2: AFM image of the photoconductive device; Figure S3: AFM image for single-laser MoS_2_ track; Figure S4: Raman spectra of MoS_2_ with characteristic peaks; Figure S5: 1D to 2D LIPSS switching caused by a defect of the film; Figure S6: AFM scan and corresponding surface contour profile of the nanostructured device.
