# Supplementary material for: Laser-Synthesized 2D-MoS2 Nanostructured Photoconductors
Source: Micromachines (Basel). 2023 May 12;14(5):1036. doi: 10.3390/mi14051036 (PMC10222115; doi:10.3390/mi14051036)
Supplement: Supplementary file 1 [file micromachines-14-01036-s001.zip › micromachines-2392485-supplementary.pdf]

micromachines

MDPI

Supplementary material

Laser irradiation parameters

Table S1. Laser scanning parameters

Figure	Liquid precursor concentration, mM	Laser intensity, MW/cm²	Scanning speed, mm/s	Comments
1a	24	0.89	9	
1b	24	0.89	10	
1c	24	0.89	10	beam polarization is along scanning direction
1d	48	0.89	19.5	
2a	24	0.89	10	
2b	24	0.89	10	
3a	48	0.51	19.5	
3b	48	0.64	19.5	
3c	48	0.76	19.5	
3d	48	0.89	19.5	

LIPSS in WS_2

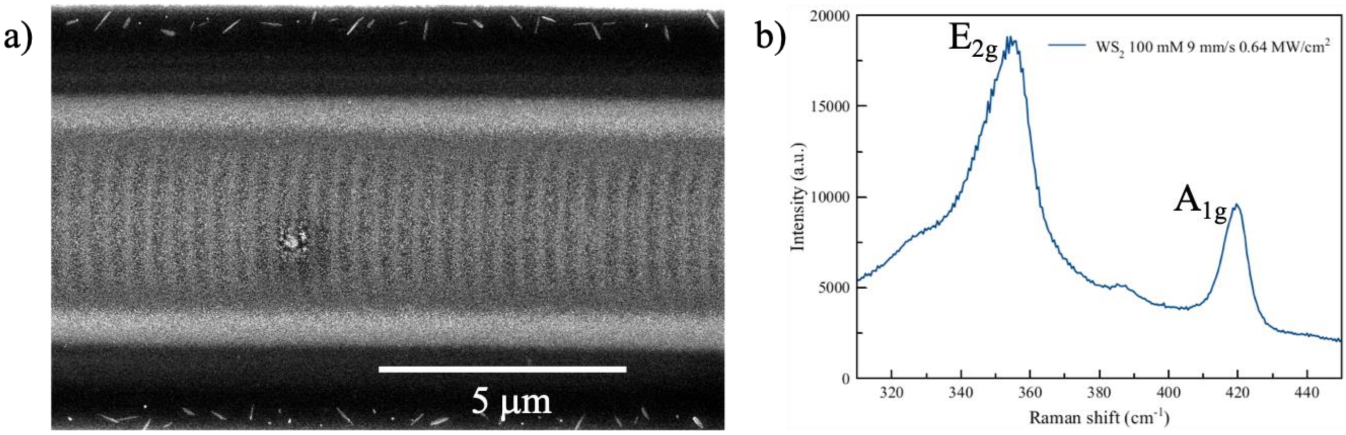

Figure S1. a) SEM image of LIPSS formation in a laser synthesised WS_2 track, using 532 nm laser radiation b) corresponding Raman spectrum showing the out-of-plane (A_{1g}) and in-plane (E_{2g}) vibrational modes. For more detailed Raman spectroscopy analysis of laser-synthesised TMDs see: O. A. Abbas, *et al.*, *Sci. Rep.* **2021**, *11*, 5211., and A.V. Averchenko, *et al.*, *Mater. Today Adv.* **2023**, *17*, 100351 (references 7, 8 in the main text).

AFM topography.

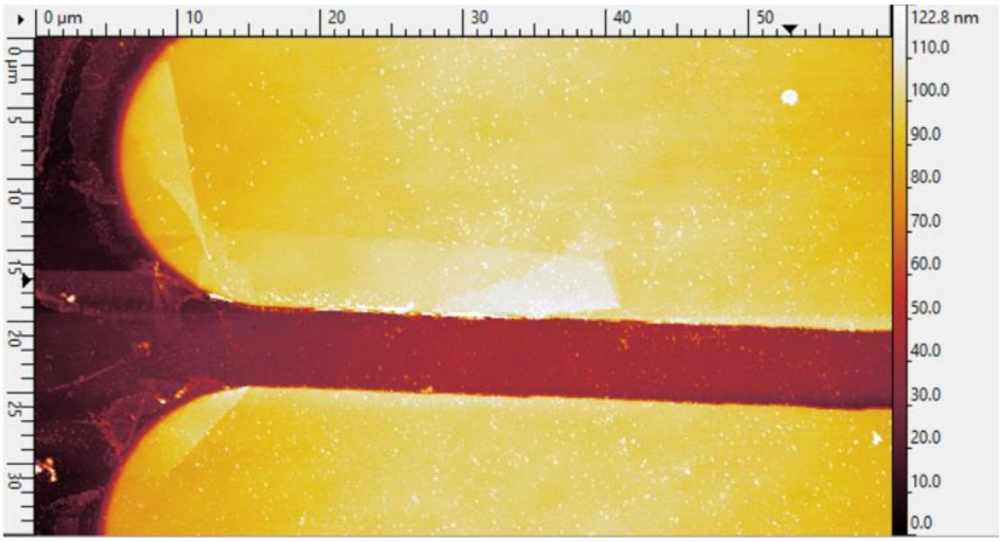

Figure S2. a) AFM image of the photoconductive device showing the two gold contacts that have been deposited either side of the film track. The channel length (gap between gold contacts) is ~7 µm.

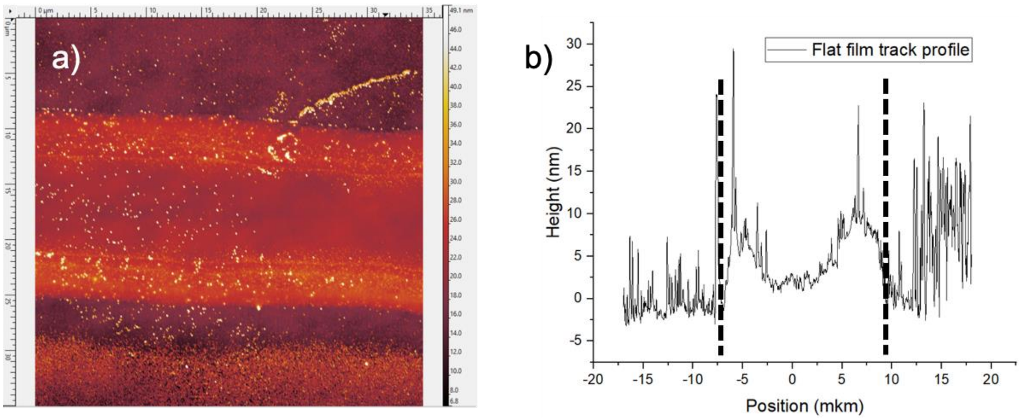

Figure S3. a) AFM image for single laser MoS_2 track exhibits a continuous film without any LIPSS features, b) Profile taken across the track (the thickness profile of the track is contained between dash lines).

Raman spectroscopy.

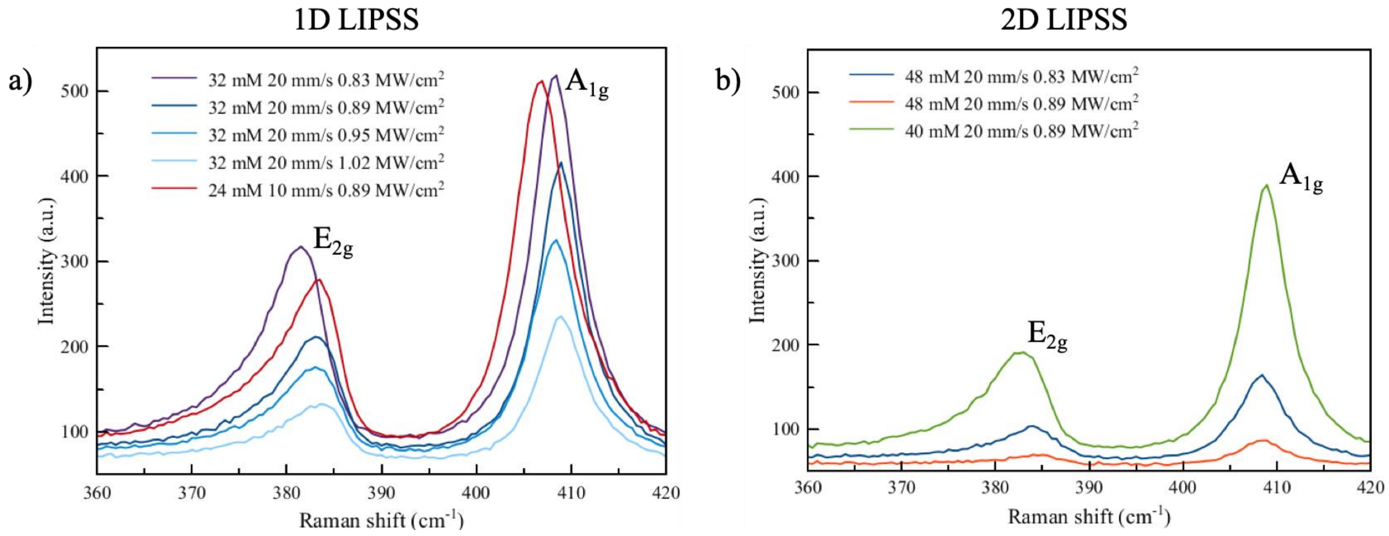

Figure S4. Raman spectra of nanostructured MoS_2 tracks corresponding to a) 1D LIPSS and b) 2D LIPSS. The Raman spectra were acquired from the centre of laser synthesised tracks, which were produced using various precursor concentrations and laser intensities (as shown in the legend of the graphs). The variety of synthesis parameters result in different film characteristics, i.e. thickness, which is reflected in the shifts of the A_{1g} and E_{2g} peaks, which correspond to out-of-plane and in-plane vibrations respectively. For more detailed Raman spectroscopy analysis of laser-synthesised TMDs see: O. A. Abbas, *et al.*, *Sci. Rep.* **2021**, *11,* 5211., and A.V. Averchenko, *et al.*, *Mater. Today Adv.* **2023**, *17,* 100351 (references 7, 8 in the main text).

SEM

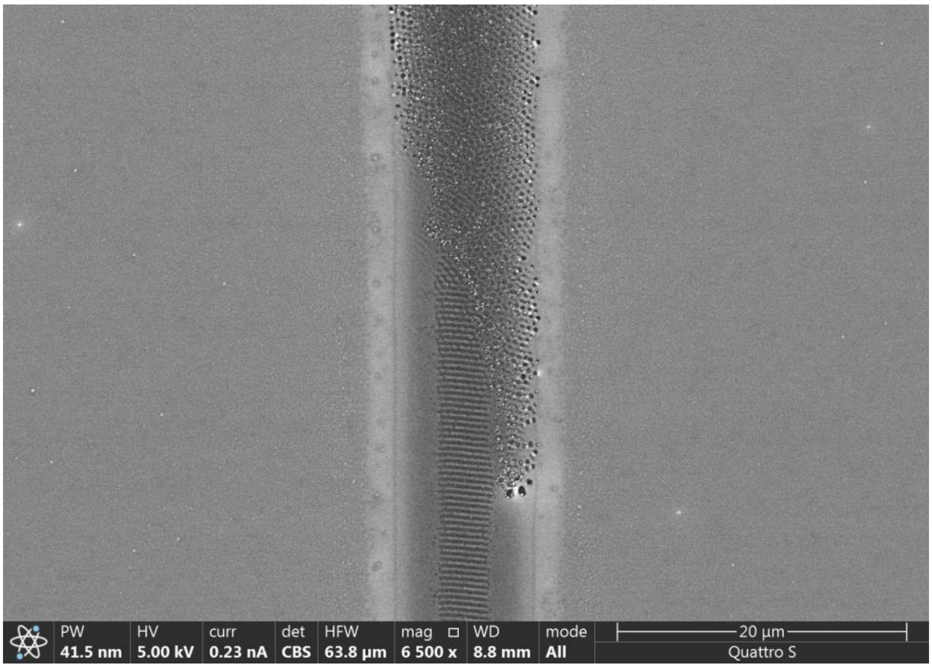

Figure S5. 1D to 2D LIPSS switching caused by a defect of the film (scanning direction is from bottom to top).

AFM scan of nanostructured photodetector

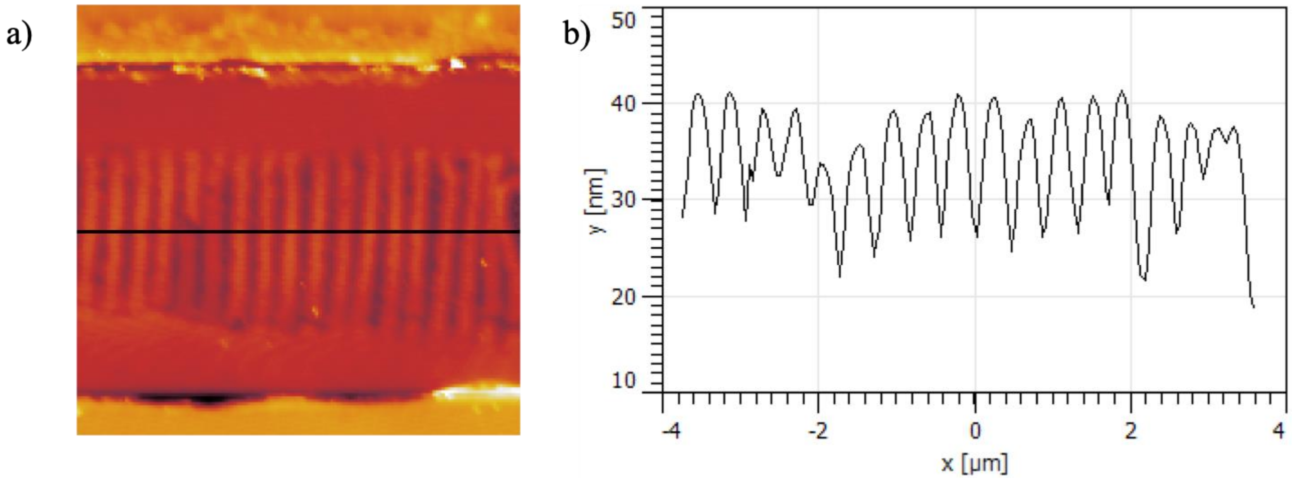

Figure S6. a) AFM scan of a section of a nanostructured photodetector consisting of MoS_2 nanoribbons, b) a topography contour extracted from the AFM image.
